# µCT scanning effects on aDNA and a multi-step workflow for archaeological petrous portions

**DOI:** 10.1371/journal.pone.0334682

**Published:** 2026-04-13

**Authors:** Lumila Paula Menéndez, Pierre Luisi, María Clara López-Sosa, Sergio Monteiro Da Silva, Laura T. Buck, Susan C. Kuzminsky, Mirsha Quinto Sánchez, Anne Le Maître, Christine Chappard, Cassandra Rios, Hans Groh, Wara Siles, Gustavo Montiel Hernández, Lorena Becerra-Valdivia, Juan Manuel Argüelles, Bernardo Yáñez, Constanza de la Fuente Castro, Camila Tamburrini, Vivette Garcia-Deister, Miguel Angel Contreras-Sieck, Sebastian Pasqualini, Paula Novellino, Candela Acosta Morano, Daniela Guevara, Daniela Mansegosa, Horacio Chiavazza, Sebastian Giannotti, Luis Tissera, Sebastián Pastor, Ivan Díaz, María Solange Grimoldi, Verónica Silva-Pinto, Ana Solari, Anne-Marie Pessis, Ramiro Barberena, Nicolás Rascovan

**Affiliations:** 1 Department of Anthropology of the Americas, University of Bonn, Bonn, Germany; 2 Laboratorio de Antropología Biológica, Escuela de Antropología, Universidad de Costa Rica, San José, Costa Rica; 3 Human Evolution and Archaeological Sciences (HEAS), University of Vienna, Vienna, Austria; 4 Instituto de Antropología de Córdoba (IDACOR), Consejo Nacional de Investigaciones Científicas y Técnicas (CONICET), Córdoba, Argentina; 5 Departamento de Antropología, Universidad Nacional de Córdoba, Córdoba, Argentina; 6 Programa de Referencia y Biobanco Genómico de la Población Argentina (PoblAr), Puerto Madryn, Argentina; 7 Institut Pasteur, Université de Paris Cité, CNRS UMR, Microbial Paleogenomics Unit, Paris, France; 8 Universidade Federal de Pernambuco, Recife, Brazil; 9 Research Centre for Evolutionary Anthropology and Palaeoecology, School of Biological and Environmental Sciences, Liverpool John Moores University, Liverpool, United Kingdom; 10 Department of Anthropology, California State University Channel Islands, Camarillo, United States of America; 11 Instituto de investigaciones Antropológicas, Universidad Nacional Autónoma de México, Mexico City, Mexico; 12 Konrad Lorenz Institute for Evolutionary and Cognition Research, Klosterneuburg, Austria; 13 Department of Evolutionary Biology, University of Vienna, Vienna, Austria; 14 Laboratoire Paléontologie Evolution Paléoécosystèmes Paléoprimatologie (PALEVOPRIM), UMR CNRS INEE, Université de Poitiers, Poitiers, France; 15 Laboratoire d’Imagerie Biomédicale, Sorbonne University, Paris, France; 16 Universidad Nacional de La Plata, La Plata, Argentina; 17 Laboratorio de Tecnologías Aditivas, Instituto de Investigaciones de Antropología y Arqueología, Universidad Mayor de San Andrés, La Paz, Bolivia; 18 Museo Nacional de Historia Natural (MNHN), La Paz, Bolivia; 19 Programa de Doctorado en Antropología, Posgrado en Antropología, Universidad Nacional Autónoma de México, Mexico City, Mexico; 20 Department of Anthropology and Archaeology, University of Bristol, Bristol, United Kingdom; 21 Linacre College, University of Oxford, Oxford, United Kingdom; 22 Dirección de Antropología Física, Instituto Nacional de Antropología e Historia, Mexico City, Mexico; 23 Coordinación Nacional de Antropología, Instituto Nacional de Antropología e Historia, Mexico City, Mexico; 24 Instituto de Ciencias Biomédicas, Facultad de Medicina, Universidad de Chile, Santiago de Chile, Chile; 25 Centro de Investigación sobre el Envejecimiento, Cinvestav-Sede Sur, Mexico City, Mexico; 26 Facultad de Ciencias, Universidad Nacional Autónoma de México, Mexico City, Mexico; 27 Department of Anthropology, University of Minnesota, Minnesota and St. Paul, United States of America; 28 Instituto Nacional de Antropología y Pensamiento Latinoamericano (INAPL), Consejo Nacional de Investigaciones Científicas y Tecnológicas (CONICET), Buenos Aires, Argentina; 29 Consejo Nacional de Investigaciones Científicas y Tecnológicas (CONICET), Buenos Aires, Argentina; 30 Museo de Ciencias Naturales y Antropológicas Juan Cornelio Moyano, Mendoza, Argentina; 31 Universidad Nacional de Cuyo, Mendoza, Argentina; 32 Laboratorio de Arqueología Histórica, Instituto de Arqueología y Etnología, Facultad de Filosofía y Letras, Universidad Nacional de Cuyo, Mendoza, Argentina; 33 Museo Arqueológico Cerro Colorado, Agencia Córdoba Cultura, Cerro Colorado, Argentina; 34 Instituto Regional de Estudios Socioculturales (IRES), Consejo Nacional de Investigaciones Científicas y Tecnológicas (CONICET), San Fernando del Valle de Catamarca, Argentina; 35 Universidad Isalud, Buenos Aires, Argentina; 36 Facultad de Filosofía y Letras, Universidad de Buenos Aires, Buenos Aires, Argentina; 37 Instituto de las Culturas (IDECU), Consejo Nacional de Investigaciones Científicas y Técnicas (CONICET), Buenos Aires, Argentina; 38 Facultad de Ciencias Sociales e Historia, Universidad Diego Portales, Santiago de Chile, Chile; 39 Programa de Doctorado en Geografía e Historia del Mediterráneo desde la Prehistoria a la Edad Moderna, Universitat de València, Valencia, Spain; 40 Área de Antropología, Museo Nacional de Historia Natural, Santiago de Chile, Chile; 41 Fundação Museu do Homem Americano (FUMDHAM), São Raimundo Nonato, Brazil; 42 Centro de Investigación, Innovación y Creación (CIIC), Universidad Católica de Temuco, Temuco, Chile; University of Szeged Institute of Biology: Szegedi Tudomanyegyetem Biologia Intezet, HUNGARY

## Abstract

The petrous portion of the temporal bone (often informally referred to as the “petrous bone”) is a key element in human evolutionary studies due to its exceptional preservation of biomolecules and morphological information. Intensive and often redundant sampling raises concerns about sustainability and long-term conservation, however. Digital recording of morphology (micro-computed and medical tomography) can preserve what destructive sampling destroys, enabling future analyses, yet there have been concerns regarding its effect on biomolecular preservation. Here, we present a systematic observational assessment of whether micro-computed tomography (µCT)—a widely used tool for digital preservation—affects ancient DNA (aDNA) integrity in archaeological human petrous portions. We analyzed 93 archaeological samples from Argentina, of which 50 were µCT-scanned prior to molecular analysis and 43 were not. We compared six commonly used molecular parameters, including endogenous DNA content, read length, cytosine deamination patterns, and mitochondrial and nuclear contamination estimates. No statistically significant differences were observed between scanned and unscanned samples across these parameters (Mann-Whitney/Wilcoxon tests, *p* > 0.05). Although mitochondrial contamination estimates were marginally higher in scanned samples (*p* = 0.051), they largely remain below the widely accepted 5% threshold for genomic analysis. Moreover, this pattern was not observed when considering nuclear contamination. Within the limits of this non-paired design, these results suggest that µCT imaging, under the scanning parameters applied here, does not introduce large or systematic bias derived from disruptions in standard aDNA preservation metrics. Building on these observations and on our collaborative experience with the shared use of archaeological samples across complementary research lines, and given that our results show that µCT imaging under appropriate scanning conditions does not significantly compromise DNA preservation, we propose a sustainable, multi-step workflow that integrates biological profiling, osteobiography, imaging, and compositional pre-screening prior to molecular sampling. This approach aims to maximize the scientific information obtained from skeletal collections while minimizing destructive practices, thereby promoting ethical and sustainable research on irreplaceable anthropological remains, and fostering interdisciplinary collaboration.

## 1. Background

Over the past two decades, advances in archaeometry—including imaging techniques and next-generation DNA sequencing—have significantly enhanced our understanding of human evolutionary history [1,2,3]. In particular, the search for optimal sources of ancient DNA (aDNA) has led to the widespread adoption of sampling the petrous portion of the temporal bone, due to its exceptional biomolecular preservation [4,5–7].

**Fig 1:**
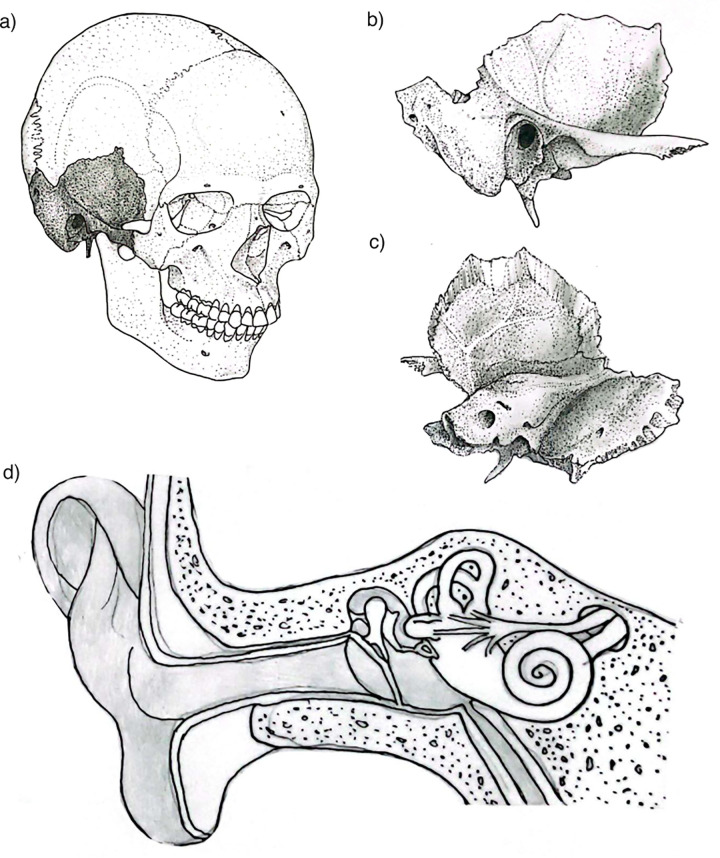
Main anatomical features of the temporal bone and petrous portion: a) human skull with the temporal bone highlighted, b) temporal bone from lateral view, c) temporal bone from internal view showing the petrous portion; d) internal detail of the petrous bone showing the location and position of the inner and middle ear. The drawings were created by Sergio Monteiro Da Silva..

Initially, teeth were considered the best skeletal source of aDNA because of their relative resistance to contamination [8,9,10]. However, recent research has shown that the cochlea within the petrous portion of the temporal bone (often informally referred to as the “petrous bone” despite not being anatomically a separate bone; Fig 1a-d) exhibits even better DNA preservation, outperforming all other anatomical structures [11,4,5–7]. More recently, despite the considerably lower preservation of ear ossicles (Fig 1d) in archaeological samples, they have been shown to yield DNA of comparable quality and complexity to that of the cochlea, leading to the recovery of similar amounts of endogenous DNA, mtDNA coverage, nuclear SNP coverage, and number of SNPs called [12]. Other studies focused on preserved ancient molecules—such as stable isotopes and radiocarbon dating—have followed this trend, favoring the petrous bone for sampling (e.g. [13,14,15]). As a result, the petrous (Fig 1c) has become the preferred target for most molecular studies, being targeted and primarily used for DNA analysis, and consequently employed in radiocarbon dating, ZooMS, and stable isotope studies when multiple analyses are conducted on the same individual or sample.

Beyond its molecular significance, the petrous bone also holds considerable value for morphological and other evolutionary studies. The cranial base and temporal bone have long been recognized as cranial regions that are less susceptible to environmental influences and preserve a strong phylogenetic signal, which are useful for reconstructing population histories [16,17,18,19,20]. More recently, the bony labyrinth within the petrous portion (Fig 1d) has been identified as an anatomical structure that reliably reflects random evolutionary changes and, as such, retains both phylogenetic and population-level signals [21,22,23,24,25]. As a result, the petrous bone has become a key resource across multiple research domains.

The growing demand for petrous bone samples, however, has not been accompanied by a sustainable research management strategy. Despite attempts to reduce damage and implement minimally invasive protocols, current practices still lead to the destruction of the cochlea, preventing detailed analysis of the bony labyrinth, and impeding the study of temporal and basicranial forms [26]. The rapid expansion of aDNA studies has led to what some researchers describe as “petrous fever”—a facet of the broader “aDNA rush” or “aDNA frenzy” [27]. This trend is mostly characterized by extensive sampling practices that undermines preservation arguments and ethical recommendations, particularly by large laboratories. These labs often operate without clearly defined research questions and instead pursue exploratory objectives, raising concerns about the long-term sustainability of such practices [27–33]. The emphasis on generating genetic data frequently comes at the expense of other valuable sources of biological information—such as morphology, histology, and isotopic analyses—which provide critical insights into past human health, diet, mobility, and cultural practices.

Neither has the momentum of the aDNA rush been accompanied by systematic efforts to digitally preserve samples prior to their destruction. One widely available approach is CT scanning, which uses X-rays and computer algorithms to generate detailed cross-sectional and 3D reconstructions of internal structures [3,34]. Two main types are commonly used: medical CT, which captures entire human bodies at resolutions of 0.5–1 mm, and µCT, which offers much finer resolution (1–100 µm) for small samples and is especially useful for applications requiring detailed structural analysis, such as studies of the inner ear. Despite its benefits for digital preservation, CT scanning raises concerns regarding the potential effects of X-ray exposure on biomolecular preservation. Since CT uses ionizing radiation, it may damage biological molecules, including DNA, and thereby compromise the integrity of ancient genetic material in bone [35].

Studies in both contemporary and prehistoric animals suggest that, when appropriate CT scanning protocols are applied, conventional μCT scanning does not reach radiation doses considered harmful for DNA [36,35,37]. Similar results have been reported for X-ray radiography of 18th-century and Bronze Age human femora [38]. In such cases, DNA degradation does not exceed the natural rate of temporal decay. These findings support the safe use of radiography and μCT scanning in bioarchaeological studies, provided that suitable imaging practices are followed, although some evidence suggests that μCT scanning may reduce collagen yield, potentially affecting the radiocarbon dating of bone samples [39].

At the same time, it is not yet clear whether these conclusions hold for archaeological human petrous bones—currently the most targeted element for aDNA retrieval—and under the specific imaging conditions of μCT scanning commonly used in contemporary biological and evolutionary anthropology. No previous study has examined archaeological human petrous bones directly even though this structure has distinctive biological and taphonomic characteristics. X-ray exposure cannot simply be inferred from studies on animal remains, soft tissues, or even other human bones. The human petrous bone’s unique microstructure and long diagenetic history may influence how radiation interacts with the tissue and how biomolecules are affected. Given the central role of the petrous portion in paleogenomic research, it becomes essential to determine whether routine μCT imaging might compromise the material that preserves the highest-quality DNA. This consideration motivated us to test these effects directly on archaeological human petrous bones.

The petrous portion of the temporal bone is uniquely dense and consistently yields exceptionally high proportions of endogenous DNA [40,6]. For this reason, even minor alterations on DNA integrity could have disproportionate implications for paleogenomic research. However, the distinct microstructure and diagenetic history of this element mean that potential effects of X-ray exposure cannot be inferred directly from studies on other skeletal elements or species. The present study therefore provides an updated observational comparison of aDNA preservation in archaeological human petrous portions that were either µCT-scanned or not scanned prior to molecular analysis, under standard µCT parameters used in biological sciences.

In this paper, we advocate for an urgent shift in the research design and management of human remains for paleogenomics, bioarchaeology, forensic science, and human evolutionary studies. To support this call, we present the results of an experiment comparing DNA preservation in 93 petrous samples from archaeological sites in Argentina (located in the Andes, Central Argentina, and Cuyo regions), spanning the Middle to Late Holocene. Some of these samples underwent μCT scanning, while others from the same assemblages did not (S1 Table). Building on these findings, we propose a sustainable workflow that maximizes the biological information derived from the petrous portion of the temporal bone, while also addressing the challenges posed by intensive sampling from skeletal collections worldwide.

Because concerns about μCT exposure directly influence sampling decisions, our results provide the foundation for the sustainable workflow we propose later in the manuscript. We also recognize that adopting such a workflow will not be equally straightforward for all research groups. In many regions, particularly across the Global South, limited access to imaging equipment, funding, and technical support makes these steps much harder to implement [41]. Even so, we think that well-resourced laboratories, especially those in the Global North that often work with collections and samples originating from the Global South, are in a position to take the lead. We believe they have both the capacity and the responsibility to begin adopting more sustainable and less destructive practices, and to help model approaches that can eventually benefit the field as a whole. Although implementing such a strategy may present obstacles, we argue that this practice will ultimately advance scientific knowledge production. While our discussion is grounded in the field of human evolutionary research, its implications extend more broadly to forensic, paleontological, and the biological sciences.

## 2. Relevance of the temporal bone and its petrous portion

The temporal bone—a thick, bilateral structure forming part of the skull’s lateral walls and base (Fig 1a–c)—is composed of four parts: the squamous, tympanic, styloid, and petrous portions ([42,43]; Fig 1a). The petrous portion is a dense, pyramidal component and the most robust of these, housing the auditory and vestibular structures (Fig 1d).

The petrous portion is positioned between the sphenoid and occipital bones and divides the middle and posterior cranial fossae. It consists of three primary surfaces: anterior/superior, posterior, and inferior (Fig 1b-c). The anterior/superior surface forms part of the middle cranial fossa and extends from the arcuate eminence to the petrous apex. The posterior surface continues into the posterior cranial fossa and contains the internal auditory meatus, which serves as a passage for the facial (cranial nerve VII) and vestibulocochlear (cranial nerve VIII) nerves. The inferior surface houses multiple foramina that transmit essential cranial nerves and blood vessels [44,45].

**Fig 2:**
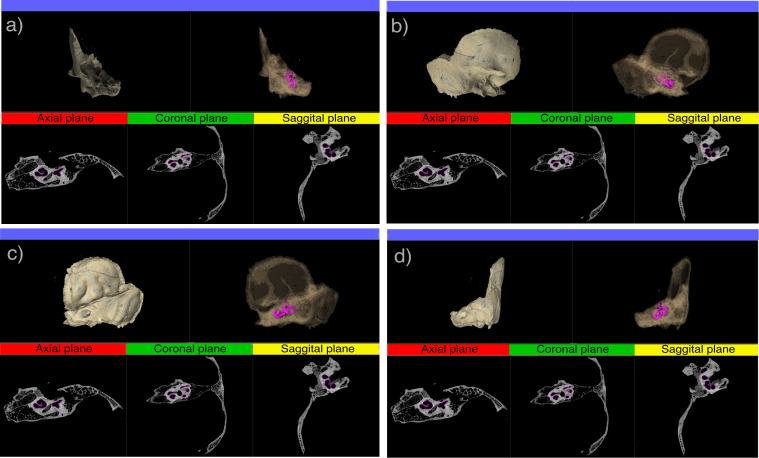
µCT scans of a temporal bone showing the segmented inner ear or bony labyrinth (highlighted in magenta) in a) anterior, b) lateral, and c) postero-lateral views. In each image a-d, the two upper panels display 3D models of the temporal bone and the inner ear (the left one with texture, and the transparent right one showing the inner ear). The three lower panels show 2D slices in the anatomical orthogonal planes indicated above: R(Red) = axial, G(Green) = sagittal, and Y(Yellow) = coronal. This Figure was created using 3D Slicer Preview Release 5.9.0 ([46]; https://www.slicer.org) and Inkscape..

Internally, the petrous portion encloses several anatomical structures, with the inner and middle ear being the ones composed of bone (Fig 1d). The inner ear, or bony labyrinth, is a highly complex structure responsible for hearing and balance. It is located in the petrous portion of the temporal bone, near its center and oriented medially and slightly anteriorly (Fig 2a-d). To illustrate its location, position, and orientation, we prepared a video showing an inner ear within a temporal bone analyzed in this work (Fig 2 [47]). The inner ear consists of the cochlea, semicircular canals, and vestibule (Fig 1d). The cochlea is a spiral-shaped organ that converts mechanical sound waves into neural signals, which are then transmitted to the brain via the auditory nerve. The semicircular canals detect rotational movements of the head, which aids in balance and spatial orientation. The vestibule plays a key role in detecting linear acceleration and head positioning relative to gravity. The middle ear is an air-filled cavity that plays a crucial role in sound transmission. It is located between the tympanic membrane and the inner ear and contains the ossicles (malleus, incus, and stapes) (Fig 1d). Other non-bony structures contained within the petrous portion include the membranous labyrinth, internal acoustic meatus, carotid canal, jugular foramen, and facial canal. Additionally, two smaller bony structures are present: the arcuate eminence and the tegmen tympani [48].

One of the most distinctive features of the petrous portion of the temporal bone is its early development and exceptional stability over time, making it highly valuable for research across multiple disciplines [40]. Fully formed by the eighth week of intrauterine life, the petrous undergoes little to no postnatal remodeling during adulthood—unlike most other skeletal elements. It is also the densest and most highly mineralized part of the human skeleton (its name derived from the Latin *petrosus*, meaning “rock-like”), traits that contribute to its remarkable preservation in both archaeological and fossil contexts. These properties not only protect the bone from post-mortem degradation but also make it one of the most reliable sources for the recovery of aDNA. Its compact structure consistently yields high quantities of endogenous DNA, making it the most sought-after skeletal element in molecular analysis of ancient humans [40,49,22,12]. In addition to its utility for molecular analyses, the petrous bone structural stability also ensures the preservation of fine morphological details, which are often lost in other skeletal elements due to taphonomic processes. This makes it an equally valuable resource for studies focused on skeletal morphology [40,21,22,23,24,25].

## 3. A proposal for a sustainable workflow in petrous bone studies: benefits and challenges

As described above, the petrous portion of the temporal bone holds exceptional informational value across multiple disciplines, making careful research design essential when planning studies involving its sampling for molecular analysis. Given this significance, we issue an urgent call to optimize workflows that maximize the preservation of biological information from this structure. Implementing such measures is crucial for safeguarding data and ensuring its availability, not only for current researchers, but also for future generations of scientists [50].

During visits to collections across different continents, some of us have encountered cases in which both petrous bones from a single individual were entirely removed—one for genetic analysis and the other for radiocarbon dating, or sometimes both for separate attempts of DNA extraction, carried out by the same or different laboratories (i.e. “double sampling”)—even though a single sample would likely have been enough [51]. Yet it remains unclear how many of these samples were digitally preserved prior to destructive analysis—likely only a small fraction. This often depends on whether principal investigators secured funding or resources for digital preservation—which is rarely the case—or collaborated with morphologists who had both access to the necessary resources and a specific interest in studying this structure, as well as the foresight to recognize the value of digitally preserving the samples.

After an entire decade of intensive petrous bone sampling for genomic research, it is imperative to reassess current practices in light of long-term sustainability and responsible resource use. The petrous portion has become the preferred skeletal element for aDNA retrieval [4,5–7], contributing to the generation of over 10,000 ancient human genomes in the past decade [52]. In fact, according to the metadata associated with the curated compendium of ancient human genomes, the Allen Ancient DNA Resource (AADR v9 [52]), aDNA data has been retrieved from petrous bone in 47% of all sequenced individuals. Importantly, the petrous bone was the only sampled bone in 96% of these cases. However, large-scale genetic data production often comes at the expense of other valuable lines of inquiry [27,30,31]. Given the finite nature of skeletal collections, the responsibilities of curatorial stewardship, the ethical considerations surrounding destructive sampling, and the rapid evolution of sampling methods, it is crucial to adopt a more balanced, multi-proxy research strategy—one that ensures paleogenetic analyses complement rather than overshadow other approaches. By embracing such a framework, the field can move toward a more comprehensive and sustainable use of the petrous bone, preserving its scientific value while continuing to deepen our understanding of past populations.

Some researchers have implemented protocols to ensure the sustainable use of information contained in petrous bones. To this end, the same petrous bone is used for multiple purposes: before it is partly pulverized to generate molecular data (genomic, radiocarbon, and isotopic), biological anthropologists perform CT and surface scans to capture its morphological information. While this sequence of operations may slow the production of genomic and isotopic data, we believe it enables researchers to fully harness the diverse information embedded in bone. Incorporating digitalization protocols prior to sampling for genomic and isotopic data, enhances research workflows and maximizes the biological information provided by multiple independent sources. Moreover, these practices foster interdisciplinary collaboration, support the integration of multiple lines of evidence on a single issue, and ensure the digital preservation of valuable morphological data for future research. In contrast, ongoing disciplinary fragmentation often results in researchers working in isolation, which can limit the potential for meaningful cross-disciplinary insights and comprehensive interpretations [53].

## 4. Testing the effects of µCT scanning on aDNA preservation in a set of petrous fragments

### 4.1. Study design and project history

This study emerged from an existing collaboration rather than from a purpose-built experimental design. In 2021, one of us (L.P.M.) contacted the lead geneticist (N.R.) knowing that he was already sampling petrous portions from archaeological individuals for a population history project focused on South American genomic diversity. After some conversations, we suggested incorporating μCT scanning before any destructive procedure so that the internal structures—especially the inner ear—could be documented under safe exposure conditions. With this goal in mind, we selected petrous portions that were as complete as possible, with minimal sediment and a high likelihood of allowing successful segmentation of the bony labyrinth. Once the μCT scans had been acquired, and because some individuals within the broader project had not been scanned, we realized that this mixed dataset provided a rare opportunity to test whether μCT scanning had any measurable effect on aDNA preservation. The analyses presented here reflect that opportunity and the practical and ethical constraints of working with archaeological human remains, where sampling cannot be repeated or redesigned once material has been processed. The dataset therefore reflects decisions made prior to the present analysis, rather than a design optimized retrospectively for hypothesis testing.

Because CT scanning relies on X-ray radiation, we sought to evaluate whether such exposure could affect the authenticity, quality, and/or contamination levels of aDNA extracted from archaeological petrous bones that were µCT-scanned and compared to others from the same archaeological sites that were not. To address this, we compared six key parameters commonly used in aDNA research: endogenous DNA content (1); average read length (2); frequency of cytosine-to-thymine (C → T) substitutions—analyzed separately for libraries that were UDG-treated (3) and those that were not (4); contamination estimates, which were based on mitochondrial DNA (mtDNA) using ContamMix (5), and nuclear DNA contamination estimates using X chromosome-based methods for individuals with an XY karyotype (6) (Table 1). Prior to scanning, petrous bones were kept inside their archival polyethylene bags, which limited direct handling and minimized the risk of contamination.

**Table 1:** Key variables for assessing ancient DNA authenticity, quality, and contamination.

Parameter	Definition
Read length	Length of the unique sequenced DNA fragments aligned to the reference genome. aDNA is highly fragmented over time, with typical lengths between 30–80 base pairs. Fragments shorter than 30 base pairs are often discarded during data processing due to its nonspecificity [54,1].
Deamination pattern (C → T)	Deamination of cytosine to uracil results in apparent C → T substitutions at 5’ ends and G → A at 3’ ends. This damage pattern is a hallmark that helps authenticate the ancient origin of DNA [55]. Ionizing radiation may also induce similar substitutions ([35,56]). Deamination damage can be erased or reduced using Uracil-DNA glycosylase (UDG) enzymatic treatment. Non-UDG libraries retain full deamination signals; half-UDG libraries preserve terminal substitutions but remove internal damage, allowing authenticity checks while improving sequencing accuracy [57]
Endogenous content	Represents the proportion of DNA in a sample originating from the target species (e.g., ancient humans) as opposed to microbial, present-day humans or environmental sources. High endogenous content is essential for effective genome reconstruction [1].
Mitochondrial contamination	Estimated using the tool ContamMix, which compares the recovered mitochondrial sequences against present-day reference panels to detect the presence of exogenous human DNA [58].
Nuclear contamination	Assessed using X-chromosome–based methods in XY individuals, where heterozygosity should be near zero due to having a single X chromosome. Elevated heterozygosity suggests contamination [59].

Because this study is based on a non-paired, opportunistic dataset derived from real-world research practices, the analyses presented here do not allow causal inference or the exclusion of subtle effects of µCT scanning on aDNA preservation. Rather, the results address whether large or systematic disruptions in commonly used aDNA quality and authenticity metrics are detectable under the scanning parameters applied. The absence of statistically detectable differences should therefore be interpreted as negative observational evidence within these limits, not as experimental proof of absence of effect.

### 4.2. Selected samples

**Fig 3:**
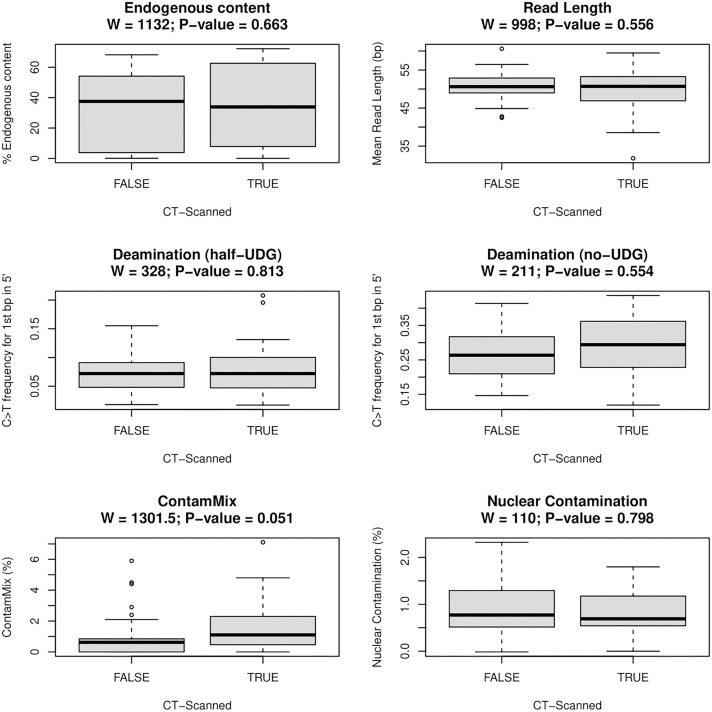
Comparison of aDNA preservation and contamination parameters in µCT-scanned (“True”; *n =* 13 for nuclear contamination; *n =* 50 otherwise) and not µCT-scanned (“False”; *n =* 18 for nuclear contamination; *n =* 43 otherwise) petrous samples: **(a)** Endogenous content: percentage of unique reads mapping to the human reference genome; **(b)** Average read length mapping to the human reference genome; **(c)** Frequency of C → T transitions at the 1st base-pair of the 5’ end of the reads for half-UDG treated DNA libraries, and for (d) non-UDG treated DNA libraries; Contamination estimates (in %) based on (e) mitochondrial DNA (with ContamMix, f) and nuclear DNA (f, only for XY individuals). *p*-values are derived from a Wilcoxon rank-sum test conducted in R [60].

We analyzed petrous bone samples recovered from Middle and Late Holocene (~ 6,000–200 y BP) archaeological contexts across North West, Central, and Central West present-day Argentina (the Andes, Central Argentina, and Cuyo, respectively; S1 Table). We selected samples that were either µCT-scanned (“True”; *n* = 50) or not (“False”; *n* = 43) prior to aDNA extraction (Fig 3). Although the samples derive from comparable archaeological contexts (i.e., the same archaeological sites, S1 Table), and they are not matched pairs (i.e., elements from the same individual), which limits our ability to attribute any observed differences exclusively to µCT scanning, the dataset provides a valuable comparative framework for assessing potential scanning effects across a diverse set of archaeological petrous portions.

Importantly, the regional distribution of µCT-scanned and unscanned samples is broadly comparable across the Andes, Central Argentina, and Cuyo regions. This balance reduces the likelihood that the observed similarities in aDNA preservation metrics are driven by geographically structured preservation conditions or region-specific laboratory histories, thereby strengthening the interpretive value of the comparative analysis (S1 Table). Moreover, scanned and unscanned petrous portions show overlapping temporal distributions, with no systematic clustering of one group in substantially older or younger periods. This temporal comparability minimizes the risk that age-related molecular degradation alone explains the observed patterns [1].

The dataset reflects the practical and ethical constraints of archaeological research rather than a purpose-built experimental design, meaning background effects related to preservation state, selection bias, temporal variation, and prior handling cannot be fully excluded. Making these constraints explicit is essential for interpreting the results within their appropriate observational scope.

### 4.3. Ethics and permits

The sampling of archaeological human remains was conducted under the necessary permits for archaeological research in Argentina (S1 File). The process of sampling and exporting the materials for ancient DNA (aDNA) analysis and CT scanning involved two steps. First, the issuance of a permit by the relevant cultural heritage authorities of the provinces where the remains were excavated and housed. Second, an export permit granted by the National Registry of Archaeological Sites, Collections, and Objects (RENYCOA), which operates under national jurisdiction to safeguard Argentina’s archaeological heritage and is responsible for authorizing the study of archaeological remains in foreign laboratories. The RENYCOA export permits for the samples analyzed in this study are: EX-2021–01946268, EX-2021–01968244, EX-2021–126859221, EX-2021–17680741, EX-2021–18124171, and EX-2021–18137399.

### 4.4. µCT scanning and aDNA processing

µCT scanning was conducted on 50 samples using a Skyscan 1176 Bruker scanner at the Faculty of Medicine, Paris Diderot University (Table 2). Up to four samples were scanned simultaneously in the chamber under the parameters presented in Table 2. These values were selected to achieve sufficient resolution for morphological analyses while minimizing exposure, following recommendations from previous studies on radiological effects on biomolecules [36,35]. Gloves and masks were worn during the placement and retrieval of the bags, and scanner components were cleaned regularly between runs to reduce potential cross-sample contamination.

**Table 2:** Description and operational values of µCT-scanning parameters applied in the present study.

Parameter	Definition	Values
Voxel size	The 3D equivalent of a pixel; it defines the spatial resolution of the scan. Smaller voxel sizes yield higher-resolution images, allowing finer anatomical details to be captured, but they require higher radiation doses. In μCT, voxel sizes are usually measured in microns (µm).	35 µm
Filter	A physical filter made of metal—typically aluminum or copper—is placed between the X-ray source and the sample to reduce low-energy X-rays. These filters, (ranging from 0.25 mm to 1 mm in thickness), reduce unnecessary radiation exposure while maintaining image quality by removing low-energy X-rays from the beam before they reach the sample.	0.5 mm aluminum
Exposure time	The duration in which each projection or slice is exposed to X-rays. Longer exposures increase image clarity by reducing noise but also raise the total radiation dose.	132 milliseconds
Voltage	The energy level of the X-ray beam, measured in kilovolts (kV). Higher voltage improves penetration of dense materials like bone but also increases radiation dose and may reduce contrast.	65 kV
Intensity	The strength of the X-ray beam, typically measured in microamperes (μA). Higher intensity increases the number of X-ray photons, enhancing image brightness and signal-to-noise ratio, but also contributes to the overall radiation dose.	393 µA

We sequenced aDNA libraries produced from these 50 scanned petrous samples and other 43 unscanned petrous bones that we used for our comparison (S1 Table). The specific procedures followed for aDNA extraction, library preparation, and sequencing are the same as those described in detail in Barberena et al. [61].

### 4.5. Statistical analysis

To assess potential differences in aDNA recovery between scanned and unscanned samples, we compared the distributions of the six parameters specified in Table 1 using non-parametric tests (the data are non-normal). Mann–Whitney U (Wilcoxon rank-sum) tests were carried out in R v4.3.2 [60]. A threshold of *p* < 0.05 was used to evaluate significance. Because the samples were not matched pairs, this approach was considered appropriate for comparing independent groups. Descriptive statistics (medians and interquartile ranges) and parameter distributions were also examined to identify possible trends not captured by significance testing.

### 4.6. Results of the experimental design

S1 Table provides a summary of the metadata for the analyzed individuals and DNA preservation results for each of the petrous samples. We did not find any statistically significant differences between the µCT-scanned and not µCT-scanned samples for any of the evaluated parameters (Mann Whitney U/Wilcoxon rank-sum test; *p* > 0.05) (Fig 3, Table 3). This suggests that, under the scanning conditions that we applied (Table 2), µCT imaging does not significantly compromise aDNA preservation, as assessed by the parameters considered here (Table 1). However, as previously noted, our opportunistic dataset allows only unpaired comparisons, and therefore does not permit a formal experimental demonstration of the absence of a scanning effect.

**Table 3:** Wilcoxon rank-sum test results comparing µCT-scanned and unscanned petrous bones across key molecular parameters. Sample sizes vary by metric: for all parameters except nuclear contamination, *n* = 50 (scanned) and *n* = 43 (non-scanned); for nuclear contamination (XY individuals only), *n* = 13 (scanned) and *n* = 18 (non-scanned). No statistically significant differences were detected for any parameter considered here (*p* > 0.05; see also Fig 3).

	U/W (Mann-Whitney/Wilcoxon statistic)	*p*-value
Endogenous content	1132	0.663
Read length	998	0.556
Deamination (half-UDG)	328	0.813
Deamination (no-UDG)	211	0.554
ContaMix	1301	0.051
Nuclear contamination	110	0.798

Read length and C → T substitution patterns in both half and non-UDG-treated libraries—commonly used indicators of aDNA fragmentation and authenticity—were similar between the two groups, providing no evidence of µCT-induced strand breaks or elevated damage. These findings align with previous studies suggesting that standard μCT protocols fall below radiation doses known to accelerate molecular degradation [36,35].

Endogenous content appeared to be very similar between the two groups (*p* = 0.663). Actually, the highest endogenous content was observed in the µCT-scanned samples. While this might seem counterintuitive, it likely reflects selection bias towards scanning better preserved bones rather than a biological effect of µCT scanning. In practice, researchers may prioritize samples that appear to be well-preserved, excluding more degraded ones from imaging workflows.

Contamination estimates based on mitochondrial data (ContaMix) were slightly higher in µCT-scanned samples (*p* = 0.051), although most (48 out of 49) remained below the commonly accepted 5% threshold for genomic analyses (S1 Table [58,62]). Although a marginal trend was detected in mitochondrial contamination, values remained below widely accepted thresholds and are best explained by sample selection rather than scanning effects. In contrast, no significant difference was observed between groups in nuclear contamination estimates (*p* = 0.798), with the highest value (2.3%) found in a sample that was not µCT-scanned.

In summary, our results provide no significant evidence that µCT scanning negatively impacts aDNA preservation, quality, or authenticity. Our analyses are not intended to substitute controlled, paired experimental studies, but to document the range of outcomes observed under realistic research conditions when µCT imaging precedes molecular analysis. Although minor trends were observed in some parameters, particularly the marginal increase in mitochondrial contamination (*p* = 0.051), they are most parsimoniously explained by differences in sample selection or handling, rather than by the scanning process itself. These findings support the integration of µCT imaging into research workflows, if appropriate protocols are followed. By doing so, researchers can digitally preserve valuable morphological data prior to destructive sampling, and contribute to the broader goal of fostering a sustainable and interdisciplinary approach to human evolutionary research.

Overall, these results have direct implications for how petrous portions are assessed, documented, and sampled in current research practices, and they form the basis for the workflow we outline below.

## 5. Proposed workflow for a sustainable petrous bone research agenda

To maximize the preservation of biological information, we propose that once access has been granted and the necessary permits to study the samples have been approved, human remains undergo a series of preliminary assessments before bone powder or fragments are collected for molecular or any other form of destructive analysis. The workflow outlined below is informed by the empirical observations presented in this study, together with existing methodological and ethical guidelines for research on human remains [63,64]. While controlled experimental studies remain necessary to evaluate subtle or dose-dependent effects of imaging on aDNA, the results presented here support the practical feasibility of integrating µCT-based digital preservation into interdisciplinary research workflows under clearly defined and explicitly stated conditions. We do not suggest that the workflow proposed here should be the full responsibility of a single individual; rather, we envision this process as a collaborative and interdisciplinary team effort.

**Fig 4:**
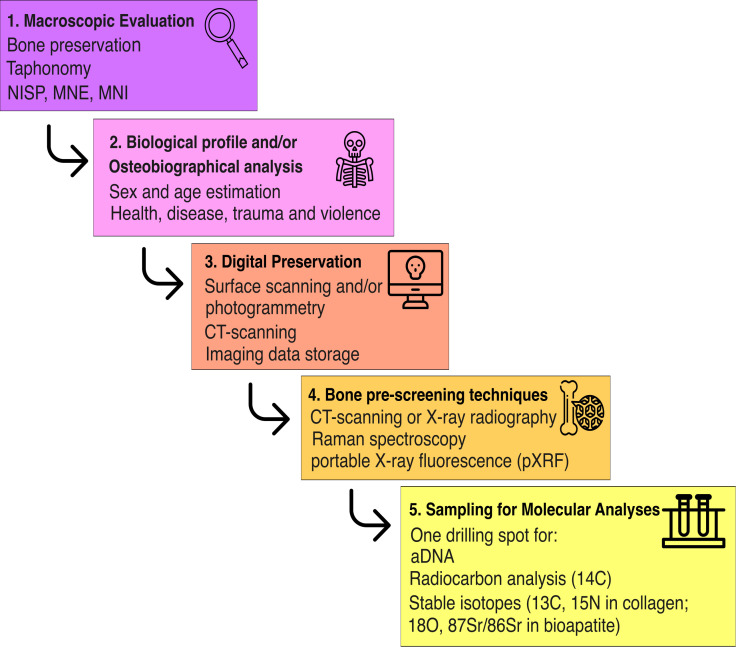
Workflow proposed for a sustainable and interdisciplinary approach to human evolution studies.

The multi-step workflow we suggest (Fig 4) is as follows:

### 5.1. Macroscopic evaluation

A detailed macroscopic assessment of bone preservation, diagenesis, taphonomy, and overall sample quality is key for making informed decisions about subsequent analyses [65,66].

### 5.2. Biological profiling and osteobiographical analysis

The first step should be a comprehensive preliminary report that provides fundamental information about the skeletal elements of each individual. This includes estimations of sex and age at death, as well as the identification of notable pathological conditions such as trauma, disease, or enthesopathies, which may open new avenues for further investigation [67,68]. Once this foundational analysis is complete, an osteobiographical assessment can be undertaken. Osteobiography involves reconstructing an individual’s life history through the study of their skeletal remains, integrating biological data with archaeological and cultural context [69].

### 5.3. Digital preservation

Either medical, or preferably, µCT scanning—complemented by surface scanning or photogrammetry—should be conducted to create permanent digital records of samples. CT scanning enables the study of internal structures, while surface scanning and photogrammetry provide complementary data by preserving surface texture information. Together, these methods complement each other effectively [34,3]. Recommendations for optimizing medical and µCT scanning parameters while minimizing DNA damage in dry bones and mummified remains can be found in Loynes & Bianucci [70], Martin- Champetier et al. [71], and Immel et al. [35]. To prevent redundant scanning, researchers should avoid repeating scans when comparable data are already available [35]. In this context, data sharing—under the responsibility of national and private institutions and their curators—and the use of public or institutional repository databases play a crucial role in promoting efficient and sustainable research practices.

Ensuring that imaging datasets are stored, governed, and shared responsibly is also central to a sustainable research workflow. While several open-access repositories—such as MorphoSource, MorphoMuseuM, Open Science Framework, or institutional digital archives—provide stable platforms for hosting µCT scans, surface models, and segmentations, the decisions about where and how to deposit these files must be guided by ethical considerations, including stewardship responsibilities and consultation with descendant or affiliated communities. For collections originating from the Global South, or from groups with specific cultural protocols, local institutions and community representatives should have a leading role in determining access conditions, permissions, and long-term digital governance.

### 5.4. Bone pre-screening techniques to assess molecular preservation

In bone-collagen radiocarbon dating and stable isotope analysis, quick and cost-effective pre-screening techniques help assess collagen preservation and guide sampling strategies by identifying morphologically insignificant bone fragments that meet the thresholds for more labor-intensive and costly downstream analyses. Common approaches include measuring nitrogen content (%N) as a proxy for collagen presence [72], near-infrared spectroscopy (NIR), a non-destructive technique [73], and X-ray fluorescence (XRF) analysis [74]. Zooarchaeology by mass spectrometry (ZooMS)—a relatively inexpensive, high-throughput technique—has also proven valuable in this context, particularly when integrated into species identification workflows [75]. Given the established correlation between collagen preservation and DNA survival [76,77,78], integrating these pre-screening techniques—alongside emerging minimally-destructive workflows—into multi-method approaches that incorporate aDNA analysis could enhance efficiency, reduce costs, and minimize destruction. Such strategies may also reduce reliance on sampling morphologically significant elements, such as the petrous bone, at the outset of molecular investigations.

### 5.5. Sampling for molecular analyses

Sampling should only proceed once steps 1–4 have been completed to ensure the most informed and responsible selection of samples. By conducting osteobiographical analysis, macroscopic evaluation, digital preservation, and XRF analysis beforehand, researchers can minimize unnecessary destruction and maximize the scientific value extracted from each sample. While steps 1–3 may be exploratory in nature, steps 4 and 5 should be conducted with a clear research question that cannot be answered using other research methods. Once a sample is deemed suitable for molecular analysis, careful consideration should be given to the sampling location and technique in order to preserve as much of the remaining bone structure as possible. CT scanning can be used to identify areas with sufficient bone density and well-preserved anatomical structures. When possible, researchers should prioritize areas that have already sustained damage or are less crucial for future morphological and histological studies [79].

By following this workflow, molecular analyses can benefit from the access to relevant contextual information while optimizing time and financial resources. Identifying the anatomical elements present for each individual allows for the selection of the most suitable human remains for sampling. Assessing diagenetic features helps determine whether a sample, or specific portions of it, is viable for analysis. CT scanning also allows researchers to identify the densest areas within a specimen, which can guide the selection of regions most likely to yield well-preserved biomolecules [80]. Additionally, understanding an individual’s biological profile and lifestyle contributes to selecting the most appropriate samples for addressing specific research questions, while providing helpful contextual information for interpreting results from molecular analyses. Moreover, CT scanning, surface scanning, and photogrammetry methods ensure the digital preservation of samples, allowing researchers to revisit their original state before the sampling occurred and for making the data accessible for future studies. X-ray fluorescence (XRF) analysis helps preserve valuable resources by preventing the unnecessary destruction of irreplaceable samples that may not meet the required compositional standards for further analysis. Finally, we recommend reducing the number of samples taken from the same individual for different analyses. Whenever feasible, tests such as radiocarbon dating, isotopes, and DNA should be performed using material from a single sampling event. CT scanning can aid this process by identifying optimal sampling areas and minimizing unnecessary damage.

## 6. Main challenges of imaging studies

We acknowledge that incorporating CT scanning into research workflows introduces additional steps into an already complex, expensive, and often time-consuming process for accessing and studying human skeletal remains. This additional step may significantly delay analyses, increase costs, and subsequent publication. Nevertheless, institutions with substantial technical and financial resources, particularly laboratories in the Global North that frequently analyze collections and samples from the Global South, are especially well positioned to initiate this change. Their access to infrastructure, funding, and expertise places them in a unique position to assume responsibility for developing and implementing more sustainable, less destructive research practices, and to set standards that may later be adopted more broadly across the discipline. To that end, here we outline the main challenges associated with this approach and propose potential solutions based on our collective experiences.

### 6.1. Challenges in accessing imaging equipment

Access to imaging equipment varies widely across the globe. In the most favorable scenarios, some institutions housing skeletal series and collections have their own µCT scanners, along with trained personnel to coordinate the logistics and conduct the scanning process (e.g., the Natural History Museum in Vienna, Austria). In contrast, other institutions are in remote areas where even medical CT scanners are unavailable within thousands of kilometers. Similar disparities exist for surface scanning and XRF, both of which depend on financial resources and trained personnel. To mitigate these challenges, researchers should allocate sufficient time to identify the most suitable conditions for imaging and collaborate with institutions that can provide access to the best available equipment and expertise. Establishing partnerships early in the research process can help secure the necessary resources and streamline logistical planning.

### 6.2. Financial constraints and potential solutions

The cost of CT scanning poses another significant challenge. With a global average of approximately $100 per scan, some research projects have sufficient funding to include CT imaging for multiple skeletons, while others have limited expenses or have not planned to allocate funding for CT scanning. Based on our experience working in countries from the Global South, alternative solutions can be explored when budgets are limited. One approach is to establish agreements with public medical institutions, universities or faculties (engineering, medical, chemical, veterinary) or collaborate with private medical facilities, which may provide access to CT scanners at reduced or no cost to the researcher.

### 6.3. Choosing between medical and µCT scanners for skeletal imaging

While medical CT scanners are more accessible, often cheaper, and allow for scanning multiple bones at once, their resolution is considerably lower than that of µCT scanners. As such, medical CT is better suited for larger anatomical structures (e.g., long bones, pelvis) and not recommended for skulls, where small structures like the inner and middle ear require higher resolution for clear visualization. However, in some cases, medical CT scanners may be the only available option. In such instances, we recommend using the highest possible spatial resolution (i.e., matrix), the smallest slice thickness, and selecting a scanning protocol that optimizes bone contrast.

### 6.4. Logistical considerations for sample transport

When imaging equipment is unavailable at the institution housing the human remains, researchers should arrange for sample transportation to a facility with CT scanning capabilities. Transporting the relevant skeletal samples requires meticulous packaging to prevent damage. We recommend using non-organic materials such as polyethylene foam, aluminum, bubble wrap, and sturdy plastic containers to ensure safe transit. Samples can be CT scanned in the same containers where they are stored (bags, boxes). In contrast, photogrammetry and surface scanning present fewer logistical challenges, as many surface scanners today are portable, and the work can typically be performed *in situ*. These methods primarily require access to electrical outlets, scanning equipment and software for image processing, making them more feasible in resource-limited settings. Photogrammetry and surface scanning require specific precautions to prevent damage or contamination of samples. This includes stabilizing samples on a clean, sterilized surface, the use of gloves and masks to avoid direct contact, ensuring that tripods, cameras, and scanners are cleaned before and after use, and applying non-invasive stabilization methods that do not introduce foreign substances. Additionally, all work should be carried out in controlled environments where dust, skin particles, or chemical residues are minimized, and proper documentation of each step is critical to guarantee sample integrity and reproducibility of results.

### 6.5. Concerns about X-ray exposure and DNA preservation

Our results presented in Section 4.6: Results of the experimental design indicate that, when appropriate protocols are followed, conventional μCT scanning—and, in most cases, standard-dose clinical medical CT—generally do not reach radiation dose levels considered harmful to aDNA [36,35]. In contrast, other X-ray imaging techniques such as Synchrotron scanning can exceed these thresholds and therefore require particularly stringent precautions [35]. Under suitable conditions, DNA degradation does not surpass the natural rate of temporal decay, supporting the safe use of CT imaging in bioarchaeological research, provided that proper scanning protocols are applied.

### 6.6. The value of a collaborative approach

A collaborative approach that brings together morphologists, bioarchaeologists, radiocarbon dating experts, archaeologists, curators, and paleogeneticists can significantly enhance sample selection by ensuring that research questions are matched with appropriate and sustainable analytical methods. Such collaboration aids in identifying well-preserved and complete samples, thereby increasing the efficiency of molecular analyses and enabling more robust interpretation of integrated datasets. As shown in Section 5: Proposed workflow for a sustainable petrous bone research agenda, our proposed workflow begins with an assessment of preservation, followed by osteobiographical analysis, selection for CT scanning, and ultimately radiocarbon dating and aDNA analysis.

## 7. Final considerations

As a multidisciplinary group of scientists specializing in morphology, paleogenetics, genomics, archaeology, radiocarbon and isotope analysis, we advocate for a responsible and sustainable approach to research, particularly when working with irreplaceable human remains. The petrous portion of the temporal bone has undeniably contributed to significant advancements in human evolutionary studies. As its demand continues to rise, however, it is critical to establish research protocols that prioritize conservation and efficiency without compromising scientific progress [27,30,31,64,81,82].

The growing ethical awareness surrounding research on ancient human remains has prompted a welcome shift toward more careful stewardship and restricted access to collections. In this context, we recommend maximizing and promoting responsible management, maximize the research potential of available materials, and promote transparency and data sharing to ensure that each study contributes meaningfully to broader scientific and ethical objectives [83,64,82]. These ethical imperatives demand methodological approaches that are not only minimally invasive, but also capable of generating the greatest possible amount of information across disciplines. With recent advances in biomolecular recovery techniques—such as the extraction of aDNA from buffer solutions applied to artifacts [84]—it is crucial to fully explore such alternatives before resorting to direct sampling of key structures like the petrous portion of the temporal bone, whose preservation is vital for both future research and heritage conservation.

At the same time, transparency in data reporting and accessibility remains a key component of responsible research practices. Efforts to link different kinds of open-access datasets, such as imaging data, archaeological metadata, isotopic results, and chronological information, can greatly enhance reproducibility and allow more integrative analyses across disciplines. However, this potential can only be realized if essential analytical parameters and metadata are consistently reported. For instance, in radiocarbon research, quality-control indicators such as collagen yield, C%, or C:N ratios are often not published, making it difficult to assess the reliability of reported ages. This issue has been highlighted in large syntheses, where a substantial proportion of key values remain unreported [85,86]. At the same time, it is important to acknowledge that not all research data can be made fully open access. In fields such as paleogenomics and human morphology, restrictions imposed by curating institutions, national regulations, agreements with stakeholders, and diverse cultural perspectives on the treatment of the dead may limit the public release of datasets [87,88,89,90]. Recent discussions have further emphasized the importance of Indigenous data sovereignty [89], as well as practical challenges such as risks of misuse [90], and unequal access to analytical infrastructure and research resources, which shapes who is able to generate, access, and reuse these datasets [29,41].

A sustainable workflow ensures that valuable biological information to multiple disciplines is not lost due to excessive destructive sampling that only favors paleogenomic and/or biomolecular analyses. By integrating macroscopic evaluation, biological profiling, osteobiographical analysis, digital preservation, collagen pre-screening techniques, and sampling for molecular analyses, researchers can maximize the analytical potential of each sample. Furthermore, promoting interdisciplinary collaboration among different specialists will facilitate data sharing, reduce redundancy in sampling efforts, and support the construction of more holistic understandings of past human lives [30,53].

Unequal access to imaging technologies and funding disparities remain major geopolitical challenges in implementing these protocols [91,92]. Collaborative efforts between research institutions and public or private medical facilities can help bridge these gaps, allowing for broader access to imaging technologies such as CT scanning, surface scanning, and photogrammetry. In particular, large and well-resourced laboratories, usually located in the Global North, are especially well-positioned, and ethically obliged, to adopt and normalize such sustainable practices. This is not only because they have greater access to funding, infrastructure, and technical expertise, but also because they frequently benefit from working with collections and human remains originating from the Global South. When most of the value extracted from these collections is concentrated in institutions far from their places of origin, there is a corresponding responsibility to minimize destructive sampling, to share data and expertise, and to contribute actively to more equitable and sustainable research practices [83]. Epistemic challenges—such as how to address inconsistencies between molecular and anatomical data, reconcile differences in timescales, and navigate hierarchies of evidence—remain to be thoroughly examined and integrated into comprehensive explanatory frameworks [91,41]. Furthermore, given the historically entrenched asymmetries that structure Global North–Global South research relations, particularly in terms of infrastructure, funding, and technical expertise, researchers based in core countries should be required to include robust plans for technology transfer and long-term scientific capacity building in the countries from which they obtain samples.

Ultimately, adopting a thoughtful scientific approach—one that emphasizes careful, methodical, and ethical research—ensures that human skeletal remains continue to inform multiple lines of inquiry while also being preserved for future generations [83,64,51,92]. The petrous portion is an invaluable resource, and its use in research should be guided by principles of sustainability, stewardship, and scientific responsibility. By refining research practices and fostering interdisciplinary collaboration, the field of biological anthropology can continue to produce meaningful insights while preserving the integrity of the subjects it studies.

Overall, our results do not show evidence of compromise aDNA preservation under the parameters and protocol applied here, even if the absence of matched pairs means differences cannot be attributed solely to scanning, and subtle effects may remain undetected. Building on this, we outline a sustainable workflow that integrates imaging, osteobiography, and compositional pre-screening before molecular sampling. This strategy promotes responsible stewardship of skeletal samples and fosters interdisciplinary collaboration, ensuring that the maximum information is obtained from each sample in the most careful and ethical way. In this context, methodological rigor must be balanced with ethical responsibility, particularly when further destructive sampling would generate marginal inferential gains at the cost of irreversible loss of material.

Future research combining paired experimental designs with observational datasets such as the one presented here will be essential for refining best practices and establishing dose-dependent thresholds for imaging-related effects.

## Supporting information

S1 File Inclusivity in global research. (PDF)

S1 Table Contextual archaeological metadata and molecular metrics of the samples included in the analysis. (XLSX)
