# Supplementary material for: µCT scanning effects on aDNA and a multi-step workflow for archaeological petrous portions
Source: PLoS One. 2026 Apr 13;21(4):e0334682. doi: 10.1371/journal.pone.0334682 (PMC13075688; doi:10.1371/journal.pone.0334682)
Supplement: S1 File — (PDF) [file pone.0334682.s001.pdf]

Inclusivity in global research

PLOS' policy on inclusivity in global research aims to improve transparency in the reporting of research performed outside of researchers' own country or community and ensures that PLOS publications reporting global research adhere to high standards for research ethics and authorship. Authors of relevant research articles may be asked to complete the questionnaire below, which outlines ethical, cultural, and scientific considerations specific to inclusivity in global research. This questionnaire may be requested when researchers have travelled to a different country to conduct research, if research uses samples collected in another country, research with Indigenous populations or their lands, or if research is on cultural artefacts. Researchers travelling to another country solely to use laboratory equipment will not normally be required to complete the questionnaire. However, the questionnaire can be requested at the journal's discretion for any submission – if you have been requested to complete this questionnaire by the PLOS journal you submitted to, please do so.

Please complete the questionnaire below and include this as a Supporting Information file with your manuscript. Note that if your paper is accepted for publication, this checklist will be published with your article in the supporting information files. Please ensure that you reference the checklist in the main body of your manuscript. We suggest adding a subsection 'Inclusivity in global research' to your Methods section and adding the following sentence: "Additional information regarding the ethical, cultural, and scientific considerations specific to inclusivity in global research is included in the Supporting Information (S1 Checklist)"

The questions have been designed to be applicable to a wide range of study types, and there are subsections for both human subjects research and non-human subjects research. If any of the questions are not relevant to your research please mark them as "N/A" as appropriate.

Ethical considerations, permits and authorship

This section is applicable to all research types.

Provide details as to who granted permissions and/or consent for the study to take place in the Methods section of your manuscript. This should include the names of **all** ethics boards, governmental organizations, community leaders or other bodies that provided approval for the study. If individuals provided approval refer to these people by their role or title but do not list their name(s).

Reported on page number: 18 (Section 4.2 "Ethical considerations and inclusivity in global research"

If there were any deviations from the study protocol after approval was obtained please provide details of these changes in the Methods section of your manuscript.

Reported on page number: There are no deviations from the study protocol.

Did this study involve local collaborators that are residents of the country where the research was conducted or members of the community studied? If you do not have any authors from said communities, please provide an explanation for this below.

Yes, this study includes multiple local collaborators who reside in the country of origin of the samples and have extensive expertise in their archaeological and cultural contexts.

Everyone listed as an author should meet PLOS' criteria for authorship and all individuals who meet these criteria should be included in the author byline, rather than the acknowledgements. For further information please see the journal's Authorship Policy.

Human subjects research (e.g. health research, medical research, cross-cultural psychology)

Did you obtain written informed consent from a representative of the local community or region before the research took place? How did you establish who speaks for the community? Details of written informed consent obtained from study participants should be reported separately in the Methods section of your manuscript.

N/A.

How did members of the local community provide input on the aims of the research investigation, its methodology, and its anticipated outcome(s)?

N/A.

When engaging with the local community, how did you ensure that the informed consent documents and other materials could be understood by local stakeholders?

N/A.

Will the findings of the research be made available in an understandable format to stakeholders in the community where the study was conducted (e.g. via a presentation, summary report, copies of publications, etc.)? Please provide details of how this will be achieved.

N/A.

Non-human subjects research using specimens/ animals collected as part of the study, or those housed in archival collections. Examples include archaeology, paleontology, botany and zoology.

Did the permission you obtained from a local authority to perform the study include an agreement on access to outputs and benefit sharing? This may include procedures to enable fair distribution of the benefits and resources arising from the research performed. Please include any details of Prior Informed Consent and Benefit Sharing Agreements obtained. These may be required by field-specific regulations, for example the Convention on Biological Diversity (CBD) and the associated Nagoya Protocol.

The permits obtained from the relevant provincial authorities did not include a formal benefit-sharing agreement as defined under the Convention on Biological Diversity or the Nagoya Protocol. In Argentina, archaeological research on human remains is regulated at the provincial level. The permits issued by the provincial governments of Catamarca, Córdoba, and Mendoza authorize the study and require that research outputs be shared with the corresponding heritage institutions. In addition, Prior Informed Consent was obtained from the Comunidad Indígena Ingamana (Catamarca), including a commitment to share results and provide copies of publications to the community. Comparable agreements for consultation, authorization, and dissemination of results were established with the Consejo Provincial Indígena (Córdoba) and with the representatives of the Huarpe communities Guaytamari and Llahué Xumec (Uspallata, Mendoza).

If the material used in your study was imported, please A) provide the year it was imported and B) indicate whether permits were obtained to import/export the materials used, C) provide details of any permits obtained. If this information is not available, please indicate this.

A) All samples were exported between 2021 and 2023.
B) Permissions for the export of samples were obtained through the Argentinean Registry of Archaeological Sites, Collections and Objects (Registro Nacional de Yacimientos Colecciones y Objetos Arqueológicos; RENYCOA-INAPL).
C) The export permits from RENYCOA-INAPL for the samples analyzed in this study are the following: EX-2021-18124171, EX-2021-18137399, EX-2020-85504578, EX-2020-84737313, EX-2020-86333220, EX-2022-35037747, EX-2022-35044700, EX-2022- 66036837, 2023-47477466, EX-2021-17680741, EX-2021-01946268, EX-2021-01968244, EX-2021-126859221.

If you used archival specimens, please state how the material used in your study was acquired by the institute it is held in and provide details of any permits obtained for the original excavations/ sample collection. If this information is not available, please indicate this.

The human remains studied here are housed at different institutions and derive from different archaeological contexts.
The samples from Catamarca are housed at Instituto de las Culturas (IDECU-UBA-CONICET) and derive from excavations conducted between 2002 and 2015 as part of the "Proyecto Arqueológico Yocavil" directed by Myriam Tarragó. All excavation permits were issued by the Dirección Provincial de Antropología, Ministerio de Cultura y Turismo, Catamarca.
The samples from Córdoba are housed at different institutions. The Gutenberg material forms part of the archival collections of the Cerro Colorado Archaeological Museum. The Cerro Colorado samples derive from an Archaeological Impact Assessment (EIA) conducted in 2018 in the context of a public works project involving the burial of the main gas pipeline, directed by Sebastián Pastor and Luis Tissera. They were restituted to the Indigenous Communities. The Dique La Viña samples originate from a public archaeology excavation project led by Pastor and Tissera in 2012, while the Carrupuchina samples, curated at the CEH Segreti, were excavated by Diego Rivero in 2012-2014. Both Dique La Viña and Carrupuchina samples are housed at Centro de Estudios Históricos (CEH-CONICET). All excavation permits for the Córdoba samples were approved by the governmental office Agencia Córdoba Cultura.
The Mendoza samples include both prehistoric and historic human remains. The prehistoric samples are part of the archival collections preserved at the Museo de Ciencias Naturales y Antropológicas Juan C. Moyano, Mendoza, Argentina. The Uspallata Valley samples were excavated in 1940, while the Mendoza Lowlands samples were excavated in 2013 by Paula Novellino, Víctor Durán, and their research team. The historic samples are housed at the Centro de Investigaciones Ruinas de San Francisco and derive from excavations conducted between 1995 and 2015 across multiple excavation seasons by the research team of the Centro de Investigaciones Ruinas de San Francisco, under the direction of Horacio Chiavazza. All Mendoza samples were recovered with permits issued by the Dirección de Patrimonio, Government of Mendoza, Argentina.

How was the potential cultural significance of the materials collected in your study to local communities considered in your research design? Were Indigenous peoples and/or local researchers and institutions involved with archaeological excavations / collection of specimens? If so, please provide a description of their involvement.

The potential cultural significance of the human remains was explicitly considered in the research design through compliance with national and provincial heritage legislation, collaboration with local researchers and institutions, and, where applicable, engagement with Indigenous communities. All samples were studied under permits issued by the relevant governmental authorities, and the research was embedded in long-standing archaeological projects led by local scholars with extensive expertise in the regions and collections involved.

In Catamarca, the research was conducted within the framework of the Proyecto Arqueológico Yocavil, with approvals from the Dirección Provincial de Antropología and RENYCOA-INAPL, and with informed consent from the Comunidad Indígena Ingamana. While Indigenous communities did not participate directly in the excavations or laboratory analyses, consent for the study of the materials was obtained, and local researchers led all fieldwork and curation activities.

In Córdoba, Indigenous representatives from the Consejo Provincial Indígena (CPI) participated as observers during the Archaeological Impact Assessment at Cerro Colorado, had unrestricted access to laboratory facilities after the excavation, and were involved in post-excavation oversight. Importantly, culturally sensitive remains were restituted to the Comechingón–Sanavirón community of Cerro Colorado and the CPI for reburial in 2023. Research activities and export of samples were authorized by Agencia Córdoba Cultura and INAPL, and access to results was explicitly agreed upon among the parties involved.

For Mendoza, the study incorporated both prehistoric and historic human remains curated in provincial institutions. Research permits were granted by the Dirección de Patrimonio (Government of Mendoza), RENYCOA-INAPL, and relevant university and national funding bodies. In the case of prehistoric samples from the Uspallata Valley, members of the Huarpe communities Guaytamari and Llahué Xumec were actively involved throughout the research process, including consultation, interpretation of results, public dissemination, and co-authorship. Community engagement was formalized through meetings, bilingual communication, and a multivocal museum exhibition developed collaboratively with Indigenous representatives. By contrast, the historic samples derive from colonial Catholic contexts in urban Mendoza, where Indigenous community involvement was not pursued due to the nature of the archaeological contexts and their documented historical use. All Mendoza-related analyses were conducted within projects that underwent institutional ethical review, including evaluation by the Institutional Review Board and the CRT Guichet Unique committee of Institut Pasteur.

PL is leading a project (Wenner-Grenn Engaged Research Grant ERG-060) on co-participation with different Indigenous organizations, including Comunidad Comehingon-Sanaviron Cerro Colorado (Córdoba) and Huarpe communities Guaytamari and Llahué Xumec (Mendoza). This project aim at discussing with the organizations all the archaeogenetics work involving human remains from the organizations' territories and carried by PL in collaboration with the Microbial Paleogenomics Unit (Pasteur Institute, France) led by NR.
Overall, the study prioritized locally grounded research practices, legal and ethical compliance, and context-specific engagement strategies, recognizing that forms of community involvement must be adapted to the historical, cultural, and archaeological characteristics of each set of materials.

If your manuscript includes photographs of human remains please indicate whether authors obtained permission from descendants or affiliated cultural communities to do so.

The manuscript does not include original photographs of human remains. It includes screenshots derived from a previously published 3D visualization video of a single petrous portion. This video was produced and made publicly available as part of an open research dataset (López-Sosa et al., 2024, *3D Visualization of the Human Bony Labyrinth within the Temporal Bone of a Subadult*, Repositorio de Datos de Investigación UNLP). The use of these screenshots is strictly illustrative, does not allow individual identification, and does not depict culturally sensitive features. The original study and dissemination of the video were conducted under the appropriate institutional, legal, and ethical approvals, including permissions from the relevant curating institutions (Museo de Ciencias Naturales y Antropológicas Juan Cornelio Moyano) and engagement with affiliated communities as required for the archaeological context.
